# Effects of Temperature on Enantiomerization Energy and Distribution of Isomers in the Chiral Cu_13_ Cluster

**DOI:** 10.3390/molecules26185710

**Published:** 2021-09-21

**Authors:** Cesar Castillo-Quevedo, Carlos Emiliano Buelna-Garcia, Edgar Paredes-Sotelo, Eduardo Robles-Chaparro, Edgar Zamora-Gonzalez, Martha Fabiola Martin-del-Campo-Solis, Jesus Manuel Quiroz-Castillo, Teresa del-Castillo-Castro, Gerardo Martínez-Guajardo, Aned de-Leon-Flores, Manuel Cortez-Valadez, Filiberto Ortiz-Chi, Tulio Gaxiola, Santos Jesus Castillo, Alejandro Vásquez-Espinal, Sudip Pan, Jose Luis Cabellos

**Affiliations:** 1 Departamento de Fundamentos del Conocimiento, Centro Universitario del Norte, Universidad de Guadalajara, Carretera Federal No. 23, Km. 191, C.P., Colotlán 46200, Jalisco, Mexico; castillo.quevedo@cunorte.udg.mx (C.C.-Q.); mfmartindelcampo@cunorte.udg.mx (M.F.M.-d.-C.-S.); 2 Departamento de Investigación en Polímeros y Materiales, Edificio 3G, Universidad de Sonora, Hermosillo 83000, Sonora, Mexico; a209205768@unison.mx (C.E.B.-G.); a213216667@unison.mx (E.P.-S.); jesus.quiroz@unison.mx (J.M.Q.-C.); teresa.delcastillo@unison.mx (T.d.-C.-C.); 3 Organización Científica y Tecnológica del Desierto, Hermosillo 83150, Sonora, Mexico; 4 Departamento de Ciencias Químico Biologicas, Edificio 5A, Universidad de Sonora, Hermosillo 83000, Sonora, Mexico; a214201767@unison.mx (E.R.-C.); aned.deleon@unison.mx (A.d.-L.-F.); 5 Departamento de Bienestar y Desarrollo Sustentable, Centro Universitario del Norte, Universidad de Guadalajara, Carretera Federal No. 23, Km. 191, C.P., Colotlán 46200, Jalisco, Mexico; edgar.zamora@cunorte.udg.mx; 6 Unidad Académica de Ciencias Químicas, Área de Ciencias de la Salud, Universidad Autónomade Zacatecas, Km. 6 Carretera Zacatecas-Guadalajara s/n, Ejido La Escondida C.P., Zacatecas 98160, Zac, Mexico; germtzguajardo@uaz.edu.mx; 7 Departamento de Investigación en Física, Edificio 3M, Universidad de Sonora, Hermosillo 83000, Sonora, Mexico; jose.cortez@unison.mx (M.C.-V.); santos.castillo@unison.mx (S.J.C.); 8 CONACYT-Universidad Juárez Autónoma de Tabasco, Centro de Investigación de Ciencia y Tecnología Aplicada de Tabasco, Cunduacán 86690, Tabasco, Mexico; fortiz@conacyt.mx; 9 Facultad de Ciencias Físico-Matemáticas de la Universidad Autónoma de Sinaloa, Culiacán 80010, Sinaloa, Mexico; tuliogax@uas.edu.mx; 10 Computational and Theoretical Chemistry Group Departamento de Ciencias Químicas, Facultad de Ciencias Exactas, Universidad Andres Bello, Republica 498, Santiago 8370035, Chile; a.vasquezespinal@uandresbello.edu; 11 Fachbereich Chemie, Philipps-Universität Marburg, Hans-Meerwein-Straße 4, 35032 Marburg, Germany; pans@chemie.uni-marburg.de

**Keywords:** nanothermodynamics, thermal population, chirality, DFT, first-principles calculations, electronic structure, Cu_13_ nanoclusters, genetic algorithm, probabilities, enantiomerization energy

## Abstract

In this study, we report the lowest energy structure of bare Cu_13_ nanoclusters as a pair of enantiomers at room temperature. Moreover, we compute the enantiomerization energy for the interconversion from minus to plus structures in the chiral putative global minimum for temperatures ranging from 20 to 1300 K. Additionally, employing nanothermodynamics, we compute the probabilities of occurrence for each particular isomer as a function of temperature. To achieve that, we explore the free energy surface of the Cu_13_ cluster, employing a genetic algorithm coupled with density functional theory. Moreover, we discuss the energetic ordering of isomers computed with various density functionals. Based on the computed thermal population, our results show that the chiral putative global minimum strongly dominates at room temperature.

## 1. Introduction

Transition-metal (TM) nanoclusters have been widely studied due to their potential applications in catalysis [1,2,3], photoluminescence [4], photonics [5], magnetism [4], chirality [6], and the design of new materials [7,8]. Cu is a 3d TM with several oxidation states [9,10], which explains its reactivity and confers many interesting physical and chemical properties [9,10]. Moreover, the high boiling point of Cu makes it compatible with high-temperature chemical reactions. Clusters are aggregates of atoms at the nanoscale size, which exhibit unusual physicochemical properties [11]. Cu clusters are particularly fascinating due to their applications in catalysis [10], light-emitting devices [12], and nanotechnology [13], despite presenting challenges such as their easy oxidation [13]. The most stable structure of small Cu clusters has been investigated by density functional theory (DFT) studies [14,15,16,17] and considering the Jahn–Teller effect [18]. In the early 2000s, Poater et al. [19] characterized neutral copper clusters (Cu*_n_ n* = 1–9) using computed chemical reactivity descriptors within the DFT framework. Later, atomic structures and reactivity descriptors of Cu*_n_* CO (*n* = 1,9) were computed and discussed [19]. Calaminici et al. reported the structure of neutral and anionic Cu_9_ clusters, employing DFT [20]. Moreover, in previous combined theoretical–experimental studies, the computed removal energies were compared with the measured photoelectron spectra in anionic Cu*_n_* (*n* = 9, 20) clusters [21], and later, the optical absorption of small Cu clusters was presented [22]. Based on their geometry and electronic structure, atomic clusters could be characterized by magic numbers [1,23,24,25] that form highly symmetric structures, for example, icosahedron (ICO) and cuboctahedron (CUB) shapes [1]. From the geometrical point of view, the first magic number that appears is 13. Experimental studies have found magic TM_13_ clusters for Fe and Ti, amongst others [1,26]. Previous theoretical studies based on the empirical potential showed that the lowest energy structures of Cu_13_ clusters were the icosahedron and the cuboctahedron [27]; those structures consist of a central atom surrounded by 12 Cu atoms. In contrast, Guvelioglu et al. [28], within the framework of DFT, found that the lowest energy structure of Cu_13_ is the double-layered structure, and in the same year, Itoh et al. [29] reported a similar double-layer structure as the putative global minimum. Yang et al. [30] explored the structural evolution of Cu*_n_* (*n* = 8–20) anions and found platelike structures. Later, larger Cu*_n_* (*n* = 20–30) clusters were investigated, and it was found that the structures are based on a 13-atom icosahedral core [31]. In previous studies, Cu_13_ was investigated because it was found to have an icosahedral structure that has a high percentage of edge and corner sites and high-index facets, resulting in increased catalytic activities [32,33,34,35]. In most cases, low-energy Cu clusters have preferentially lower symmetry structures [36], although some present distorted structures [36,37]. Although there are many studies on Cu clusters, the chirality of Cu_13_ clusters has not been discussed. In general, chirality plays a decisive role in biological activity and life processes [38]. Remarkably, chiral nanoclusters have attracted attention because they have applications in chiral materials with novel properties [39,40]. Previous theoretical studies on PtPd co-doped silicon clusters reported chiral and fluxional low-energy structures [41]. Recently, Kong et al. [42] reported propeller-like chiral AIE copper (I) clusters with exciting properties. However, clusters’ properties depend on their putative global minimum and low-energy structures, considering achiral and chiral structures. Hence, we need to know the distributions of isomers at different temperatures [43]. The lowest- and low-energy geometries, composition, and temperature [43,44,45] of the ensemble determine all the properties of a cluster at temperature T, i.e., its electronic, structural, vibrational, and optical properties, as well as its chemical reactivity. Moreover, the atomic structure is the first level at which it is possible to manipulate the macroscopic properties of a cluster [1].

In this study, intending to elucidate the lowest- and the low-energy structures of neutral Cu_13_ clusters at temperature T, we explored their free energy surface by employing a genetic algorithm coupled to DFT. We computed the probability of occurrence of each particular isomer, employing nanothermodynamics for temperatures ranging from 20 to 1500 K. Our findings show that the putative global minimum is a chiral structure at room temperature. Moreover, we computed the transition state (TS), i.e., the enantiomerization energy for temperatures ranging from 20 to 1300 K, for interconversion of a pair of enantiomers (Plus, P, and Minus, M). Our computations showed that enantiomerization barriers led to persistently chiral structures and enabled the complete separation of enantiomers at room temperature [46,47]. The remainder of the manuscript is organized as follows. Section 2 provides the computational details and a brief overview of the theory and the algorithms used. The results and discussion are presented in Section 3. We discuss the low-energy structures, the effect of the DFT functionals on the energetic ordering of isomers, and the origin of the slight 0.41 kcal/mol energy differences. We analyze the interconversion energy barrier between P and M enantiomers, the effects of the temperature in the energy barriers, and the thermal population. Conclusions are given in Section 4.

## 2. Theoretical Methods and Computational Details

All geometrical structures were optimized locally without imposing any symmetry; the self-consistent field procedure was performed with a convergence criterion of 10^−6^ a.u. energy, maximum force, and maximum displacement convergence were set to 10^−6^ Ha, 0.002 Ha/Å, and 0.0005 Å, respectively. All calculations were performed using the Gaussian suite code [48], employing the Becke’s hybrid three-parameter [49,50] exchange–correlation functional in combination with the Perdew and Wang GGA functional PW91, [51,52]; this combination is known as the B3PW91 exchange-correlation functional. The B3PW91 has been employed in other studies of the reactivity of copper clusters with good performance [53,54]. Hybrid functionals, including a portion of Hartree–Fock exchange, have shown a superior performance [53,55,56]. We employed the LANL2DZ double-ζ-quality with effective core potential (ECP) [57] and the Ahlrichs-type triple-ζ-quality extended-valence def2-TZVP basis set [58]. The LANL2DZ basis set is used for transition metals due to its low computational cost [59]. With the aim of refining the optimization and the energies, we used an Ahlrichs-type triple-ζ-quality extended-valence def2-TZVP basis set [58,60] that is more accurate for transition metals [61], despite its considerably higher computational cost [62]. In this study, dispersion corrections were considered through the D3 version of Grimme’s dispersion [63] as implemented in the Gaussian code. In a previous work, the effect of the dispersion corrections on the structural and energetic properties of Be_4_B_8_ and Be_6_B_11_^−^ clusters was studied, and it was found that the energetic ordering of isomers can change when the dispersion is considered [43,47]. Transition states are discarded through a vibrational analysis, making sure that the reported structures are true energy minima. Calculation of the Gibbs free energy properties of a Cu_13_ cluster requires an exhaustive and systematic sampling of the free-energy surface with the aim of finding all possible low-energy structures [43,47,64]. Foremost, the search for the global minimum in atomic clusters is a complicated task mainly due to the increase in the degrees of freedom of a molecule with the increase in the number of atoms; as a consequence, the number of local minima increases exponentially with the number of atoms. Moreover, the calculated total energy of the cluster demands a high level of quantum mechanical methodology to produce reliable energies. Despite that, several algorithms coupled with DFT have been employed to search for the lowest energy structures on the potential energy surface of atomic clusters, such as the kick methodology [65,66,67,68,69,70,71,72,73,74,75,76,77] and genetic algorithms [43,78,79,80,81]. Our computational procedure to elucidate the lowest energy consisted of a hybrid genetic algorithm called GALGOSON [43,47]. GALGOSON employs a multi-step and multi-level search strategy in which optimizations are first performed with the LANL2DZ basis set and, in a second step, energy refinements are made using the def2-TZVP basis set. The generation of the initial population took into account 2D and 3D structures [73,78], with an initial population of 650 random structures for the Cu_13_ cluster; the algorithm was stopped when the putative global minimum persisted for five generations [43,47]. Chemical bonding was examined using the adaptive natural density partitioning (AdNDP) method [47,82]. AdNDP analyzes the first-order reduced density matrix and recovers Lewis bonding (1c–2e or 2c–2e, i.e., lone pairs (LPs), or two-center–two-electron bonds) and delocalized bonding elements (associated with the concept of electron delocalization).

In this study, the fundamental thermodynamic properties such as enthalpy H(T) and entropy S(T) and Gibbs free energy dependent on temperature were computed within the framework of nanothermodynamics [43,47,83,84] through the partition function Q described in refs. [43,85,86] or any standard text relating to thermodynamics [87,88]. The total partition function Q is the product of the q_trasn_, q_rot_, q_vib_, and q_elec_ [86,89] computed under the rigid rotor, harmonic oscillator, Born–Oppenheimer, ideal gas, and particle-in-a-box approximations [43,47]. The thermal populations P(T) at absolute temperature T or the so-called probability of a particular isomer is computed with Equation (1):

(1) $$P\left(T\right)=\frac{e^{-{\beta \Delta G}^{K}}}{\sum e^{-{\beta \Delta G}^{K}}}$$

where $\beta =1/k_{B}T$, $k_{B}$ is the Boltzmann constant, T is the temperature, and ∆G^k^ is the Gibbs free energy of the kth isomer. Equation (1) establishes that the distribution of isomers among energy levels is a function of energy and temperature [43,86,90]. 

## 3. Results and Discussion

**Figure 1:**
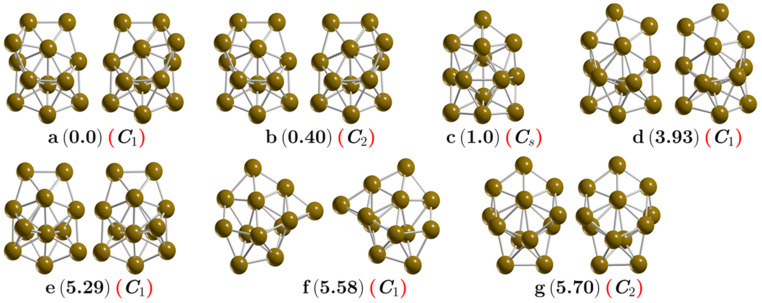
(Color online) The chiral lowest energy structure is depicted in (**a**), the chiral low-energy structures are depicted in (**b**,**d**,**e**–**g**) whereas the achiral structure is depicted in (**c**). Those structures were optimized at the PW91-GD3/def2TZVP level of theory. The first letter indicates the isomer, the relative Gibbs free energies in kcal/mol appear in round parentheses computed at 298.15 K, and the point group symmetry in red round parentheses. The isomers, represented in (**a**,**b**), differ only in molecular symmetry. The isomer with C_2_ symmetry has a Gibbs free energy equal to RTlnσ less than the non-symmetric C_1_ isomer. The RMSD between the isomer with symmetry C_1_ and the isomer with symmetry C_2_ is 0.0014. All atomic XYZ coordinates are given in Supplementary materials.

**Figure 2:**
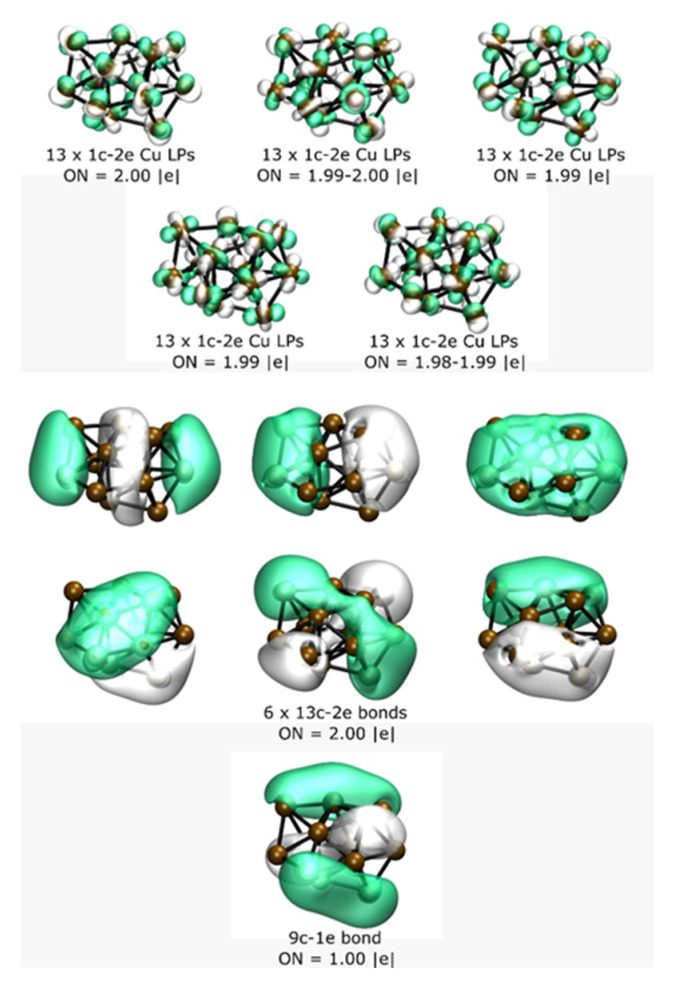
(Color online) Results of the AdNDP analysis of the lowest energy isomer of the Cu_13_ system.

The most important low-energy structures of a neutral Cu_13_ cluster optimized at the B3PW91-GD3/def2TZVP level of theory found in this study are shown in Figure 1. At room temperature, the isomers depicted in Figure 1a,b contributed to 94% of the molecular properties in a Boltzmann ensemble; thus, almost all molecular properties were due to those isomers. Additionally, they are chiral structures. The putative chiral global minimum is depicted in Figure 1a with symmetry C_1_. These are bilayered structures composed of a shared pentagonal bipyramid interspersed with a distorted hexagonal ring with a Cu atom capping one of its faces and two Cu atoms capping the other face of the hexagonal ring, in good agreement with similar structures [28,91,92]. The pentagonal bipyramid interspersed with the hexagonal ring is built with 12 Cu atoms. One more Cu atom caps the pentagonal bipyramid; this capping Cu atom is responsible for the chirality of the Cu_13_ cluster. Our calculated Cu–Cu bond length on the putative chiral global minimum is 2.432 Å, in good agreement with other reported DFT calculations of a Cu–Cu dimer of 2.248 Å [93] and also with an experimental bond length of 2.22 [94,95], slightly above 5.3% the experimentally determined value. The calculated vibrational frequency of Cu_13_ was 60 cm^−1^, whereas the computed vibrational frequency of the Cu–Cu dimer was 245 cm^−1^, again in good agreement with the experimental value of 265 cm^−1^ [95]. We also explored the higher multiplicity of quartets and found that the lowest energy structure lay 20.5 kcal/mol above the doublet putative chiral global minimum energy structure. The second structure that was higher in free energy lay at 0.40 kcal/mol at room temperature; it was also a bilayered structure, similar to the putative global minimum, but with C_2_ symmetry. Iwasa et al. [96] reported a similar double-layer structure as a putative global minimum with C_2_ symmetry, but without considering the temperature. One of our previous studies showed that these tiny Gibbs free energy differences are derived from rotational entropy [47]. The C_1_ and C_2_ symmetry clusters adopted a hollow layered structure. The following higher energy isomer lay at 1.0 kcal/mol at room temperature and was an achiral buckled-biplanar (BBP) structure with Cs symmetry, which agrees with previous work [97]. At room temperature, its contributions to the molecular properties were less than 6%. The average bond length on isomer BBP was 2.432 Å, similar to the average bond length of the chiral putative global minimum. Next, higher energy structures lay 3.9 kcal/mol above the chiral putative global minimum, and their average bond length was 2.444 Å, slightly larger than the average bond length of 2.432 Å of the putative global minimum. This also appeared as a bilayered chiral structure with a shared hexagonal bipyramid interspersed with a hexagonal bipyramid. The following higher energy structure lay 5.29 kcal/mol above the putative minimum global. It was a bilayered structure consisting of 12 atoms, with 1 atom capping one of its faces. It is depicted in Figure 1e. Structures located at higher energy than 5.5 kcal/mol above the putative global minimum are depicted in Figure 1f,g. Those structures also adopted a layered structure with no interior atoms, with similar morphology to that of low-energy isomers. These two structures did not contribute to the molecular properties in the studied temperature range. The Cu_13_ cluster low-energy structures preferentially adopted morphologies of bilayered structures rather than highly symmetric 3D structures. In contrast, Au_13_ clusters prefer planar structures due to relativistic effects [36]; therefore, further studies are needed to investigate why bilayered structures in the Cu_13_ cluster are energetically preferred. For the Cu_13_ cluster, the icosahedron structure is not energetically favorable in the temperature range examined, which is consistent with previous work where the authors did not consider the temperature [1]. In this study, the icosahedron structure was located at 24.6 kcal/mol above the putative global minimum at room temperature. To understand the bonding situation in the chiral putative global minimum structure, we performed an AdNDP analysis; the results are shown in Figure 2. This analysis revealed the presence of 5 sets of 13 1c–2e bonds with occupation numbers (ONs) between 1.98 and 1.99 |e|, i.e., lone pairs (LPs) corresponding to the fully filled 3d shell in each Cu atom. The bonding in this cluster was then due to the 4s shell electrons for which the bonding pattern, as revealed by AdNDP, consisted of 6 sets of 13c–2e completely delocalized bonds, plus a 9c–1e bond corresponding to the unpaired electron which, as shown in Figure 2, was mostly delocalized in the peripheral atoms of the cluster.

### Energetics

Temperature drastically affects the Gibbs free energy of the isomers; therefore, in a molecular ensemble (collection), the energetic ordering of isomers changes. Besides, from a theoretical point of view, the energetic ordering can also change when computing energies using different levels of theory [43,98]. To gain further insight into the energetic ordering of the low-lying isomers, we optimized the low-lying energy structures employing three more DFT functionals: TPSS, [99] PBE, [100], and BP86 [50] with def2-TZVP [58] basis set. The purpose was to ascertain the origin of the slight 0.41 kcal/mol differences (below the chemical accuracy of 1 kcal/mol) in the relative Gibbs free energy (Table 1) and that these are not due to numerical errors, algorithmic approximations, integration grids, or functional and basis set dependence, to name a few. The relative energies computed at B3PW91-D3/def2-TZVP, TPSS-D3/def2-TZVP, PBE-D3/def2-TZVP, and BP86-D3/def2-TZVP are shown in Table 1; Columns third through fifth show electronic energy, electronic with zero-point energy, and Gibbs free energy at 298.15 K at the B3PW91-D3/def2-TZVP level of theory. Columns sixth to ninth show Gibbs free energy at 298.15 K at TPSS-D3/def2-TZVP, PBE-D3/def2-TZVP, and BP86-D3/def2-TZVP levels of theory, respectively. A more detailed analysis of the results in Table 1 shows that the relative electronic energy of the four chiral low-energy isomers labeled in Table 1 (a, b, c, d), with symmetry C_1_, C_1_, C_2_, and C_2_, respectively, is zero, considering the ZPE; also, the relative electronic energy is zero. In contrast, the relative Gibbs free energy at 298.15 K shown in the fifth column is 0.41 kcal/mol. The relative Gibbs free energy at 298.15 K for the TPSS, PBE, BP86 DFT functions, between the putative global minimum and the second isomer, is also 0.41 kcal/mol. This Gibbs free energy difference does not depend on the functional employed, as shown in Table 1. At temperature T = 0, the total energy of an isomer is the electronic energy plus ZPE. If the temperature increases, entropic effects start to play, and Gibbs’s free energy determines the global minimum. At any temperature T, the isomers, represented in Figure 1a,b, differ only in molecular symmetry. The isomer with C_2_ symmetry has a Gibbs free energy equal to RTlnσ less than the non-symmetric C_1_ isomer. Here R is the universal gas constant, T temperature, and σ is the symmetry number. The symmetry number appears in the denominator of the molecular rotational partition function [43,47,101,102]. This implies that the less symmetric isomers at finite temperature are more thermodynamically stable than the more symmetric ones due to the energy factor given by RTlnσ. The factor becomes zero at T = 0 and increases linearly with temperature. Figure S1 (Supplementary materials) shows the RTlnσ factor as a function of temperature and for different symmetry numbers. For our optimized low-energy isomers with C_2_ symmetry, the symmetry number is 2, thus, the Gibbs free energy at 298.15 K with and without symmetry will differ by 0.41 kcal/mol regardless of the DFT method. This value is higher at high temperatures and with higher symmetry numbers. For example, the benzene molecule with D_6h_ symmetry has a symmetry number 12, the Gibbs free energy at 298.15 K with and without symmetry will differ by 1.47 kcal/mol, which is greater than the chemical precision. Here, we call this the effect of the symmetry number on the Gibbs free energy and on the thermal populations at temperature T; the symmetry number appears when identical atoms are considered indistinguishable and are determined solely by the point group symmetry of the molecule. We emphasize the importance of symmetry in calculating thermal populations at absolute temperature T or the so-called population probability or relative populations, hence the molecular properties. For example, the melting temperature for a symmetrical molecule is higher than a non-symmetrical molecule; moreover, the activation energy barrier could be higher when we consider a non-symmetrical molecule in the calculation of the transition state. The energy computed at different theoretical levels influences the energy distribution of the isomers and, as a consequence, the Boltzmann weights. For the four DFT functionals used in this study, the energy ordering is preserved, although differences in the energy between the isomers occur; for each DFT functional, the main contributors to any molecular property of Cu_13_ are always the chiral isomers depicted in Figure 1a.

## 4. Enantiomerization Energy of a Pair of Enantiomers of the Cu_13_ Cluster at Finite Temperature

**Figure 3:**
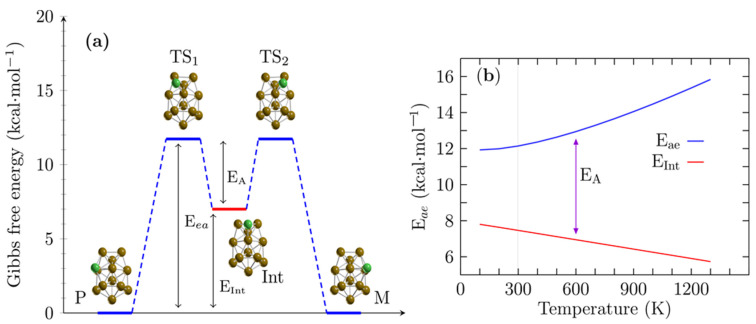
(Color online) (**a**) shows the energy profile of a chemical reaction (route A) with two symmetric transition states (TS1, TS2) and one intermediate (Int) for the interconversion between the lowest energy P and M enantiomers (see video of intrinsic reaction path in Supplementary materials). The enantiomerization energy (E_ea_) was 12.14 kcal/mol at room temperature. In contrast, the intermediate structure lay at 7.13 kcal/mol (E_Int_) above the putative global minimum P and M structures; thus, the activation energy in the interconversion from the intermediate (Int) to the M or P structure was 5.0 kcal/mol, at room temperature. (**b**) displays the enantiomerization energy (E_ea_) depicted by a blue-solid line. The relative energy of the intermediate structure (E_Int_) with respect to the putative global minimum is depicted by a red solid line. The activation energy barrier (EA) between the intermediate structure and the P or M structure is indicated by the violet arrow and is the difference between E_ea_ and E_Int_ for temperatures ranging from 100 to 1300 K. The green and copper-colored spheres represent the copper atoms. Green represents a Cu atom that is moving; the chirality is due to this atom.

The process in which one enantiomer in a pair is converted into the other is known as enantiomerization; enantiomers each have an equal probability of occurrence, and the same energy. The enantiomerization energy or activation energy at temperature T defines the configurational stability. In certain cases, a low enantiomerization energy is undesirable [103]. Two reaction mechanisms compete for the interconversion from P to M structures, and the shape of the energy barriers (or IRC) is similar to the inverted double-well potential [104] (see the two videos in Supplementary materials for the intrinsic reaction paths, called route A and route B). Figure 3a shows the reaction mechanism for the interconversion between P and M structures for route A, which proceeds via a two-step mechanism consisting of two symmetric steps with only one intermediate. Figure 3a depicts the transition states TS1 and TS2, the intermediate (Int), and the putative lowest energy pair of enantiomers P and M. The energy of enantiomerization was 12.15 kcal/mol, whereas the activation energy for the interconversion of the intermediate to P/M structures was 5 kcal/mol at room temperature. The intermediate state was located at 7.13 kcal/mol above the putative chiral lowest energy structure. The structures of the TS1 and TS2 states are depicted in Figure 3a. They appeared to be bilayer structures composed of a shared pentagonal bipyramid interspersed with a distorted hexagonal ring. The green atom represents the Cu atom that caps one edge of the pentagonal bipyramid and is responsible for the chirality of the Cu_13_ cluster. The intermediate state structure for the same bilayer presented 12 Cu atoms and the green Cu atom caps one of the faces in the pentagonal bipyramid. Figure 3b shows the enantiomerization energy E_ae_ depicted by a solid blue line. The relative energy of the intermediate, E_Int_, for the putative global chiral structures is depicted by a solid red line. As the temperature increased, the enantiomerization energy increased almost linearly. In contrast, the relative energy of the intermediate with respect to the putative global minimum decreased linearly, implying that the inverted double-wall became energetically greater. The activation energy for the interconversion between the intermediate and the M structure was 5 kcal/mol at room temperature; this increased linearly, from 4 kcal/mol at a temperature of 100 K to 9.5 kcal/mol at a temperature of 1200 K. As a consequence, the probability of interconversion from P to M decreased as the temperature increased. In contrast, at low temperatures, the enantiomerization energy trend reached a minimum, whereas the relative energy of the intermediate increased; thus, the energy activation for the interconversion of the intermediate to the P/M states tended to be smaller. These results suggest that at high temperatures, the enantiomerization barrier energy increased, and the intermediate state energy became more significant, stabilizing chirality and allowing the separation of enantiomers at room temperature [46]. To elucidate the behavior of the interconversion from P to M structures; We computed the reaction rate constants based on Equation (2) (Eyring equation) that used the activation barrier ΔG between the putative global minimum P/M structures and the transition state and did not consider the tunneling effect. The Eyring equation relates the rate constant to temperature and the activation free energy.

(2) $${k=k}_{0}\frac{K_{B}T}{h}e^{\frac{-\Delta G}{\mathrm{RT}}}$$

In Equation (2), k is the rate constant, $k_{0}$ transmission coefficient that in the absence of other kinetic data is set to 1, $K_{B}$ is Boltzmann constant, T is the temperature, h is the Planck constant, R is ideal gas constant, and ΔG is the activation energy barrier. We consider the rate-determining step in the overall reaction is the rate of interconversion between P/M and intermediary structures, and it is the slowest step; besides, its high activation energy characterizes it. (The activation energy barrier ΔG is computed with the statistical thermodynamics). The height of the activation energy barrier at room temperature for interconversion between P/M and intermediary structures in route A is 12.14 kcal/mol, which leads to a rate constant of 7.84 × 10^3^ 1/s, whereas the activation energy barrier at 900 K is 14.0 kcal/mol, which leads to a rate constant of 7.47 × 10^9^ 1/s. This show that the rate constant increases at high temperatures, and it agrees with the thermal populations where the contribution of all isomers is less than 10% at high temperatures. We also have to consider that the melting point for copper is 1358 K, thus around this temperature, the glass state will dominate. Regarding dispersion, if it is not considered, the energy barriers tend to increase. For ease of comparison, Table 2 shows the values of the two similar reaction mechanisms A and B, taking into account the D3 dispersion of Grimme. Energetically, the reaction mechanism of route B is not considerably different from that of route A, as we can see in Table 2.

The E_A_ between the intermediate and the P or M structure for route A and taking D3 Grimme’s dispersion into account was 5.0 kcal/mol, as we can see in the first line of Table 2, whereas the E_A_ for route B was 4 kcal/mol. In contrast, the values of E_A_s computed without dispersion for both routes A and B, in the second line of Table 1, were 5.97 and 4.28 kcal/mol, respectively. Based on these results, the effect of the dispersion was to lower the energy barriers in the interconversion of the chiral Cu_13_ cluster. Moreover, the reaction mechanism with the highest probability to occur was route A, because it had lower energy barriers than route B. Figure S2 shows the energy profile of a chemical reaction with two symmetric transition states (TS1, TS2) and one intermediate (Int) for the interconversion between the lowest energy P and M enantiomers for route B, and for ease comparison, the Figure S2 shows the energy profile of a chemical reaction for route A.

## 5. Relative Population of Cu_13_ Cluster at Finite Temperature

**Figure 4:**
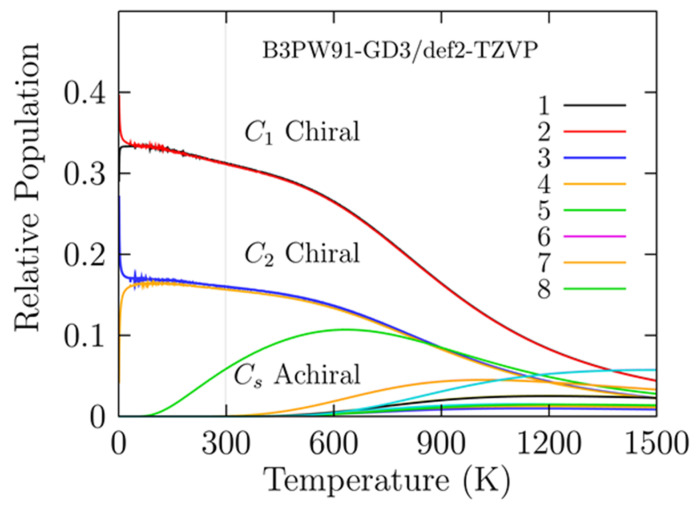
(Color online) Probability of occurrence for all isomers at temperatures ranging from 20 to 1500 K. The red and black solid lines depict the probability occurrence of the putative chiral global minimum with symmetry *C_1_* and strongly dominate in all ranges of temperatures. The bulk melting temperature of copper is 1358 K [105]; thus, our results below this temperature are consistent. Probability of occurrence or thermal population computed with DFT functionals: TPSS, PBE, and BP86 are displayed in Figure S3.

In chemistry, physics, and biology, the lowest energy structure and all the low-energy structures near the global minimum are crucial because all molecular properties are statistical averages derived from the ensemble of molecular conformations [43]. The probability of occurrence of each particular isomer is depicted in Figure 4 for the Cu_13_ cluster. It was determined by employing Equation (1) and temperatures ranging from 20 to 1500 K at the B3PW91-D3/def2TZVP level of theory. Figure 4 shows the probability of occurrence considering all chiral and achiral structures. The analysis of these results led to an interesting observation. The pair of enantiomers that appeared as the putative global minimum at temperature 0 K was strongly dominant in the temperature range from 20 to 1500 K. Moreover, there were no solid–solid transformation points in any temperature ranges, which means no interchange of dominant low-energy structures at high temperatures. A closer inspection of Figure 4 shows that the decay of probability of occurrence of the pair of enantiomers with symmetry C_1_, depicted by a red solid line, is closer to linear rather than exponential for temperatures ranging from 20 to 600 K. Above 600 K and up to 1500 K, the decay is exponential. At 300 K, the chiral structure has a probability of 32%, whereas the second isomer located 0.4 kcal/mol above the putative global minimum has a probability of 16%. The above discussion shows that all molecular properties of the Cu_13_ cluster are attributed to the chiral putative global minimum. The probability of occurrence of chiral isomers is shown in Figure 1b, located at 0.41 kcal/mol above the putative global minimum and depicted by blue and yellow solid lines in Figure 4. The probability of occurrence for structures with C_1_ and C_2_ symmetries showed similar behaviors but different values; however, the molecular properties are attributed to only one pair of enantiomers with symmetry C_1_. The probability of occurrence for the achiral structure, which is shown in Figure 1c, located 1 kcal/mol above the putative global minimum, is depicted by a green solid line in Figure 4; it started to increase around a temperature of 120 K, and at room temperature, it is has a probability of 5%. At 700 K, the highest probability of occurrence was reached, corresponding to 12%; above this temperature, up to 1500 K, it started to decrease. Note that above 800 K and up to 1200 K, the achiral structure with C_s_ symmetry and the putative global minimum structures with C_2_ symmetries coexisted. The Boltzmann ensemble was composed of an equal mixture of M and P enantiomers; thus, the chiral properties were null in all ranges of temperature, i.e., the Cu_13_ cluster did not exhibit properties such as vibrational/electronic circular dichroism. In ranging temperatures from 1200 to 1500 K, all isomers coexist with less than ten percent probability. To wit, all isomers are equally populated for hot temperatures or when the temperature increases to large values. The bulk melting temperature of copper, 1358 K [105]; thus, we must consider that the anharmonic effects become strong at high temperatures [43]. From the thermal population, we consider the entropic-temperature term has a small effect on the Cu_13_ cluster distribution of isomers on the scale of temperature, as shown in Figure 4.

## 6. Conclusions

We explored the potential and free energy surface of the neutral Cu_13_ cluster with an efficient cascade-type algorithm coupled to DFT. We found that the putative global minimum was a pair of enantiomers that strongly dominated at room temperature. Our findings show that the chirality exhibited by the Cu_13_ cluster emerged from the Cu atom capping a face of the core Cu_12_ cluster. We showed that for the interconversion between P and M structures, two similar reaction mechanisms were possible. Both of them closed in their energy barriers and proceeded via two symmetric steps. The energy of enantiomerization and the energy barrier between the intermediate and the P/M structures increased as the temperature increased. We computed the reaction rate constants based on the Eyring equation; our findings show that, at high temperatures, enantiomerization is favored. The entropic–temperature term did not significantly influence the energy barriers; thus, they are mainly composed of enthalpic energy. Regarding Grimme’s dispersion D3, this lowers the energy barriers, i.e., in route B, the EA decreased by 7% (from 4.3 to 4.0 kcal/mol). We showed that the pair of enantiomers with C_1_ symmetries strongly dominated at room temperature revealed by the thermal population. Hence, at body temperature, all the molecular properties were attributable to those structures. For each DFT functional (B3PW91, TPSS, PBE, and BP86) used in this study, the thermal population and energetic ordering of the isomers are preserved, although differences in the energy between the isomers occur; the main contributors to any molecular property of Cu_13_ are always the chiral isomers. The bonding in the lowest energy chiral Cu_13_ cluster is due to the 4s shell electrons for which the bonding pattern, as revealed by AdNDP, consisted of 6 sets of 13c–2e completely delocalized bonds, plus a 9c–1e bond corresponding to the unpaired electron. Future work will focus on the computation of UV-vis absorption of the Cu clusters employing Boltzmann weighted spectra, comparing it with a single UV spectrum of the putative global minimum.

## Figures and Tables

**Table 1:** Relative energy in kcal/mol of the low-lying isomers depicted in Figure 1 labeled from a to g, employing various density functionals: B3PW91, TPSS, PBE, and BP86, with the def2TZVP basis set. Point group symmetry (Symmetry), electronic energy ($\varepsilon_{0}$), electronic energy plus zero-point energy ($\varepsilon_{0}{+\varepsilon }_{\mathrm{ZPE}}$), and Gibbs free energy (ΔG) computed at room temperature. The tiny values of relative Gibbs free energy (0.41 kcal/mol) differences among isomers labeled a, b, c, and d and computed at 298.15 K are due to the rotational entropy, which depends on the point group symmetry.

Isomers Labeling (Figure 1)	Symmetry	Functionals					
		B3PW91			TPSS	PBE	**BP86**
		$\varepsilon_{0}$	$\varepsilon_{0}{+\varepsilon }_{\mathrm{ZPE}}$	ΔG	ΔG	ΔG	ΔG
a	C_1_	0.0	0.0	0.0	0.0	0.0	0.0
b	C_1_	0.0	0.0	0.0	0.0	0.0	0.0
c	C_2_	0.0	0.0	0.40	0.40	0.42	0.40
d	C_2_	0.0	0.0	0.41	0.41	0.42	0.41
e	C_s_	0.90	0.93	1.0	0.72	0.55	0.86
f	C_1_	4.71	4.59	3.93	5.60	3.85	3.56
g	C_1_	4.71	4.59	3.94	5.60	3.85	3.56

**Table 2:** Values of enantiomerization energy (E_ea_), relative energy of the intermediate (E_Int_), and activation energy (E_A_) for two reaction mechanisms, A and B, taking or not considering D3 Grimme’s dispersion. The inclusion of dispersion lowers the energy barriers, i.e., in B the E_A_ decreased by 7% (from 4.3 to 4.0 kcal/mol).

		Route A			Route B	
Level of Theory	E_ea_	E_int_	E_A_	E_ea_	E_int_	E_A_
B3PW91-D3/def2TZVP	12.2	7.1	5.0	14.8	10.8	4.0
B3PW91/def2TZVP	12.3	6.4	6.0	15.6	10.3	4.3

## Data Availability

Data are contained within the article.

## Supplementary Materials

The following is available online, Figure S1: (Color online) shows the RTlnσ factor as a function of temperature and for different symmetry numbers. For our optimized low-energy isomers with C_2_ symmetry, the symmetry number is 2; thus, the Gibbs free energy at 298.15 K with and without symmetry will differ by 0.41 kcal/mol regardless of the DFT method, Figure S2a: (Color online) Figure (a) shows the energy profile of a chemical reaction (route A) with two symmetric transition states (TS1, TS2) and one intermediate (Int) for the interconversion between the lowest energy P and M enantiomers, Figure S2b: (Color online) Figure (a) shows the energy profile of a chemical reaction (route B) with two symmetric transition states (TS1, TS2) and one intermediate (Int) for the interconversion be-tween the lowest energy P and M enantiomers, Figure S3: (Color online) Probability of occurrence or thermal population for all isomers at temperatures ranging from 20 to 1500 K computed with DFT functionals: (a) B3PW91; (b) TPSS; (c) PBE; (d) BP86. The influence of functional over the thermal population is not significant. The red and black solid lines depict the probability occurrence of the putative chiral global minimum with symmetry C_1_ and strongly dominate at room temperature. The bulk melting temperature of copper is 1358 K; thus, our results below this temperature are consistent and describe correctly the Cu_13_ system. Video S1: Cu_13_ route A, Video S2: Cu_13_ route B.

## Sample Availability

Samples of the compounds are not available from the authors.
